# Genetic assessment of the value of restoration planting within an endangered eucalypt woodland

**DOI:** 10.1038/s41598-023-33720-z

**Published:** 2023-04-21

**Authors:** Natalie L. Rosser, Anthony Quinton, Huw Davey, David J. Ayre, Andrew J. Denham

**Affiliations:** 1 Centre for Sustainable Ecosystem Solutions, School of Earth, Atmospheric and Life Sciences, University of Wollongong, Wollongong, NSW Australia; 2 Independent Researcher, Wollongong, NSW Australia; 3 Science, Economics and Insights Division, NSW Department of Planning and Environment, Parramatta, NSW Australia

**Keywords:** Population genetics, Conservation biology, Restoration ecology, Ecology, Genetics

## Abstract

Assessment of woodland restoration often focusses on stand demographics, but genetic factors likely influence long-term stand viability. We examined the genetic composition of Yellow Box (*Eucalyptus melliodora*) trees in endangered Box-Gum Grassy Woodland in SE Australia, some 30 years after planting with seeds of reportedly local provenance. Using DArT sequencing for 1406 SNPs, we compared genetic diversity and population structure of planted *E. melliodora* trees with remnant bushland trees, paddock trees and natural recruits. Genetic patterns imply that natural stands and paddock trees had historically high gene flow (among group pairwise *F*_*ST*_ = 0.04–0.10). Genetic diversity was highest among relictual paddock trees (*H*_*e*_ = 0.17), while diversity of revegetated trees was identical to natural bushland trees (*H*_*e*_ = 0.14). Bayesian clustering placed the revegetated trees into six genetic groups with four corresponding to genotypes from paddock trees, indicating that revegetated stands are mainly of genetically diverse, local provenance. Natural recruits were largely derived from paddock trees with some contribution from planted trees. A few trees have likely hybridised with other local eucalypt species which are unlikely to compromise stand integrity. We show that paddock trees have high genetic diversity and capture historic genetic variety and provide important foci for natural recruitment of genetically diverse and outcrossed seedlings.

## Introduction

In many parts of the world, woodland ecosystems have been extensively cleared for agriculture and urban development, often leaving only small remnants. As a result, trillions of dollars are spent on ecological restoration and revegetation planting programs each year to restore biodiversity to degraded landscapes^1–3^. The goal of restoration is to create a self-supporting ecosystem that is resilient to adversity^4^, but the effectiveness of ecological restoration programs is dependent upon a suite of population genetic issues that influence the success of ecological restoration and revegetation planting programs^5–7^. First, the level of population subdivision among remnant or revegetated populations has implications for the level and distribution of genetic diversity, and the likelihood that restoration will exacerbate inbreeding or outbreeding and may produce inbreeding or outbreeding depression^8–11^. In the long term, natural genetic subdivision for any species will reflect the dynamics of pollen and seed dispersal and patterns of extinction and colonisation. However, in restoration programs, fragmentation will initially be determined by patterns of planting and consequently by potentially atypical gene flow (e.g.^11^). If there are high levels of subdivision and low gene flow among natural stands, restoration planning should consider the likelihood of site-specific adaptation in sourcing material for transplantation^12^. Second, remnant or revegetated populations may lack sufficient or appropriate genetic diversity as a result of genetic drift, founder effects, inbreeding and inappropriate sourcing of material for transplants. Fitness can be reduced in inbred offspring either because of loss of beneficial heterozygous traits, or by the expression of recessive deleterious traits, and can include reduced seed crops, smaller seeds, poor seedling survival and lower capacity to respond to environmental change (reviewed in^13^). Restoration programs should typically aim to avoid elevated inbreeding due to small stand sizes and/or sourcing seed or seedlings from small numbers of closely related individuals. Such programs should also ensure that revegetation programs have at least natural levels of genetic diversity because it is positively associated with population fitness in the short-term, and adaptive capacity and resilience to environmental change in the long-term^14,15^. Third, many species that are targets for woodland restoration have been shown to hybridize with native and exotic species^16–20^ which can be a problem for restoration programs since first or later generation hybrids may have lower viability than pure-bred individuals^16^, and hybridization will also limit the value of restoration programs if conserving pure gene pools is a priority^21^. It is important to note however, that a range of studies have argued the benefits of utilizing first generation hybrids in restoration programs if they possess beneficial traits such as drought tolerance or elevated resistance to pests or climate change^22–24^.

Eucalypt woodlands are a key component of the Australian landscape and were once widespread in southern Australia. However, large areas have been extensively cleared since European settlement, and in many landscapes where clearing has reduced the extent of remnant woodlands, remnant paddock trees are a key resource for woodland restoration^13,25^. Eucalypts have a mixed mating system, but are predominantly outcrossed via insect pollination with outcrossing rates typically between 0.44 and 0.96^26^. Interestingly, the degree of outcrossing within a single tree can vary from the top down due to pollinator foraging patterns as they move through the canopy^27^. While eucalypt pollen can travel up to 2 km^28^, more typical dispersal distances are around 200 m^13,29,30^. These characters imply that even moderate spatial isolation will increase the likelihood of selfing or inbreeding for paddock trees and remnant stands and that populations will display evidence of subdivision. Planning or evaluating the success of restoration programs is further complicated by the fact that eucalypts can sometimes hybridize^16,17^, though the likelihood of hybridization can vary dramatically with a range of factors including the genetic distance between potentially hybridizing taxa^31^, their relative and absolute density, and pollen dispersal distances^16,32^.

In eastern Australia, the White Box-Yellow Box-Blakely’s Red Gum Grassy Woodland ecosystem (known as Box-Gum Grassy Woodland) is critically endangered and has been the target of restoration programs that have been largely unassessed (but see^13,33,34^. Broadhurst’s study^13^ using genetic (microsatellite) data to assess the diversity and mating systems of small numbers of scattered and revegetated *Eucalyptus melliodora* (Yellow Box) trees is an important exception. Broadhurst^13^ collected samples from five restoration sites each within 200 km of Canberra in the Australian Capital Territory. Despite considerable among-site variation, both in the design of the restoration plantings and proximity to paddock trees, that study highlighted the importance of paddock trees as sources of genetic diversity and as pollen sources for seed on revegetated trees^13^. Broadhurst^13^ also found considerable differentiation among paddock and planted trees and concluded that many were not of local provenance. We set out to build on Broadhurst’s work by evaluating, from a genetic perspective, the success of the restoration program in Warrumbungle National Park (WNP) where there is a set of *E. melliodora* plantings that were carried out over approximately 10 years to supplement a small number of remnant paddock trees that are potentially isolated from surrounding natural stands and that have formed foci for some natural regeneration. The restoration program is located within a 500 ha valley that had been extensively cleared for pastoral activity and grazed from the 1900s until the 1960s. Although we have observed in situ seed set and recruitment in the valley area, it is not known whether there is sufficient genetic diversity to maintain populations in the long term, or whether inbreeding is likely to constrain their persistence. Moreover, plantings are thought to have been locally sourced, but this has not been confirmed genetically, nor is anything known of the genetic make-up of either natural or revegetated stands, their mating systems or extent of genetic connectivity throughout the system.

Our specific objectives were to: (a) assess and compare the genetic differentiation and mating systems of natural stands surrounding the restoration area with that of surviving paddock trees, recruits and planted stands to estimate levels of connectedness; (b) determine whether paddock trees, recruits and planted trees capture similar levels of genetic diversity to the natural stands; and (c) use these data to infer the provenance of planted trees and to test for evidence of hybridization.

## Methods

### Study system

This study focused on Yellow Box (*Eucalyptus melliodora*) in Box-Gum Grassy Woodlands in WNP. Box-Gum Grassy Woodlands are critically endangered and are protected by Australian Commonwealth^35^ and NSW state legislation^36^. Due to the occurrence of these woodlands on fertile soils, they were extensively cleared for agriculture, and intact remnants are now extremely rare. Current estimates indicate that only 405,000 ha of the woodland ecosystem remain (~ 10%), with few high-quality remnants anywhere across its former range^37^. These woodlands are characterised by a dominance or prior dominance of White Box (*Eucalyptus albens*) and/or Yellow Box (*E. melliodora*) and/or Blakely’s Red Gum (*E. blakelyi*) trees. Box-Gum Grassy Woodland was previously abundant within the central valley of WNP (31°17′S, 149°00′E), but extensive land clearing led to the widespread removal of trees prior to the establishment of the reserve. Scattered relictual *E. melliodora* paddock trees remain in the central valley. These trees were often solitary and were distributed broadly across the valley.

Warrumbungle National Park covers an area of 23,312 ha on the north-west slopes of NSW, covering the western end of the Warrumbungle Range. A series of revegetation projects were conducted within the central valley of the reserve from 1983 to restore the previously abundant Box-Gum Grassy Woodland. The seeds for these plantings are believed to have been sourced from existing trees within the reserve. However, the number of paddock trees used or how broadly seeds were collected across the valley, is not known.

### Eucalyptus melliodora sampling

**Figure 1:**
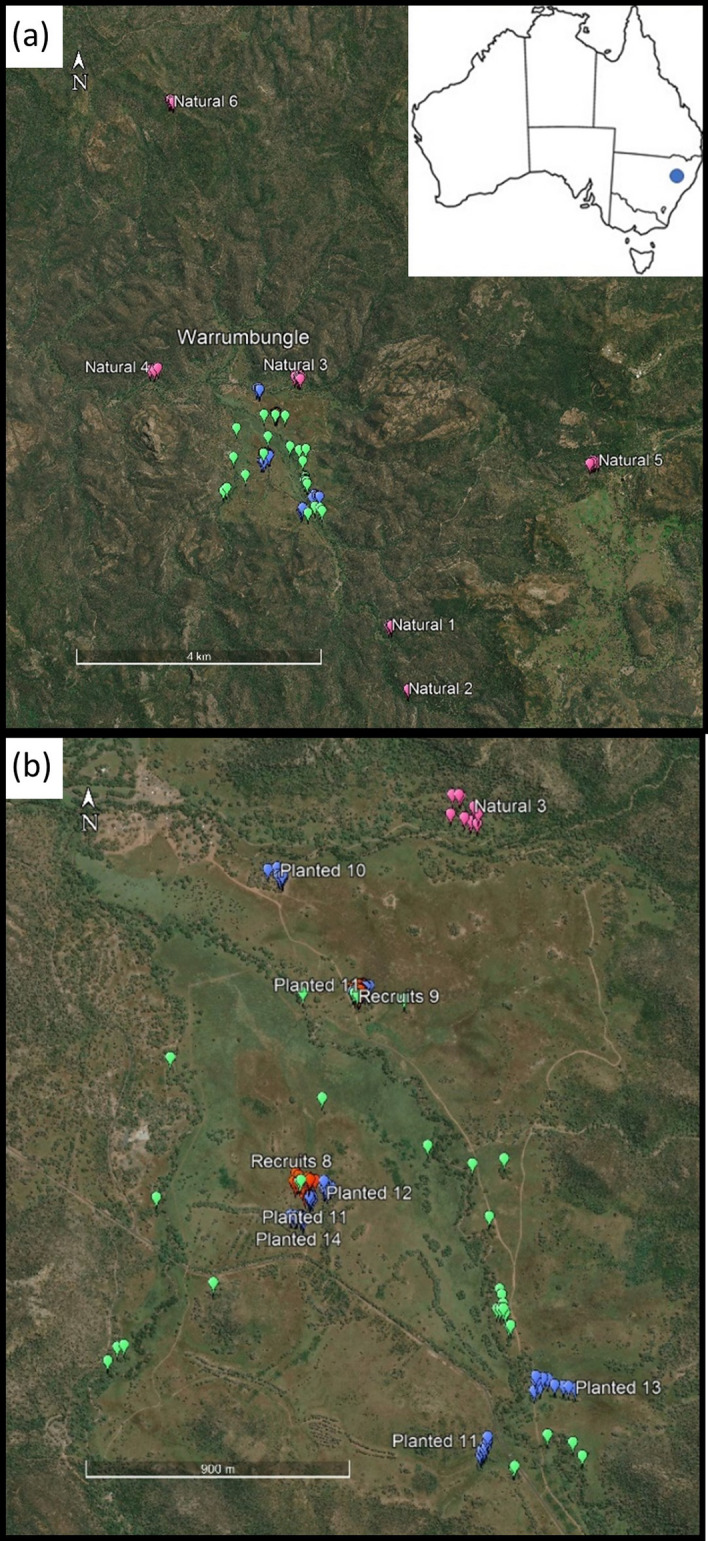
Location of sampled *Eucalyptus melliodora* plants in Warrumbungle National Park. Different colours represent different population types: Natural populations—pink; Paddock trees—green; Planted trees—blue; Recruits—orange. (**a**) Overview showing the sample locations of natural populations and the previously cleared central valley where planting occurred. The insert shows the location of the study site within NSW, Australia. (**b**) Close up of the central valley showing the sampled planted populations (grouped by planting year), the two groups of recruits and relictual paddock trees. Map created using Google Earth Pro 7.3.4.8642 (www.google.com/earth/versions/#earth-pro).

We collected a total of 221 leaf samples of *E. melliodora* trees from 14 sites across the central valley and the uncleared valley sides (Fig. 1). We selected samples from six remnant populations on the edge of valley (n = 60; termed “natural stands”); from approximately half of the paddock trees in the valley (n = 36; termed “paddock”); from two populations of recruits, each one growing near one of the paddock trees (n = 48; termed “recruits”); and from five populations of planted trees that were planted at different years in the restoration project (n = 77; termed “planted”).

We selected samples from remnant populations on the valley sides at a distance > 1 km from plantings on the uncleared slopes of the valley or separate to the central valley, with a preference for larger (likely older) trees, to increase the probability that these trees pre-date the germination of planted trees and hence provide a better reference for remnant *E. melliodora* within the region. Relictual paddock trees were distinguished from planted trees by comparing aerial photos taken prior to the establishment of the reserve (Supplementary Fig. S1) with contemporary imagery. Paddock trees were easily relocated in the cleared central valley as they are large (20–30 m tall) and often have associated woody debris, while planted trees were much smaller than paddock trees (6–15 m tall) and were generally planted in rows, often with adjacent timber posts. The five planted populations were planted in different years, with population 11 planted in 1995, population 12 in 1998 and population 14 planted in 1992, while the dates that the other two populations (10 and 13) were planted are unknown. Recruits were smaller still (0.5–5 m tall) and occurred in scattered locations, often in a ‘shadow’ surrounding adult paddock trees. Field identification was done by one of us with relevant training and experience (AJD). Voucher specimens were lodged at the Janet Cosh Herbarium at the University of Wollongong with identification formally confirmed by Patricia Nagle (WOLL12125 *E. albens* and WOLL12124 *E. melliodora*).

### DArT genotyping

We used DArTseq services (Diversity Arrays Technology, Canberra) for both DNA extraction from leaf material, and sequencing of SNPs (Single Nucleotide Polymorphisms) using a high-density microarray developed for eucalypts^38,39^. Extraction of genomic DNA was conducted in accordance with a modified CTAB protocol produced by DArT.

Complexity reduction was conducted through a *Pst*I/*Taq*I based method developed by^38^, using enzymatic breakdown to select for more active genomic regions and remove repeat sequences. Next Generation Sequencing (NGS) was used to detect SNP polymorphisms across the genome of each sample against a library developed for eucalypts^38^ detecting dominant, biallelic SNP loci. Polymorphic SNP loci were sequenced across the genome all leaf samples, using the DArTseq microarray at a ‘high intensity’ run. Sequencing was carried out on an Illumina HiSeq 2,500 using 75-cycle single end reads. Raw reads were processed using DArT's proprietary variant calling pipeline, DArTsoft-14.

### Data analysis

We filtered the primary dataset at a stringent level to ensure only high-quality markers were retained, and therefore genotypes were accurate. Loci were filtered in DARTR^40^ using the following filters: secondaries removed, read depth ≥ 10, call rate ≥ 0.9, minor allele frequency ≥ 0.01, reproducibility rate ≥ 0.99, monomorphic loci removed and individuals with 20% missing data were removed. In addition to the excluding loci with a reproducibility rate of < 0.99, we also included 10 duplicate samples in the sequencing run (i.e. 10 individuals were sequenced twice) to check the reliability of the sequencing results. For each duplicate we compared the sequencing result at each locus (0, 1, 2 or ‘– ‘ for no result) and counted the number of times this result differed between the replicates, then divided this by the total number of loci.

To analyse population structure, we calculated pairwise *F*_*ST*_ in DARTR^40^, and conducted a Principal Components Analysis (PCA) in Adegenet^41^ together with a Bayesian cluster analysis in the software *ParallelStructure 2.3.4* on the CIPRES portal^42^ to identify the number of genetic clusters in the dataset. We used the admixture model of ancestry with correlated allele frequencies, a burn-in length of 80,000 followed by 120,000 MCMC replicates after burn-in, and we conducted 5 iterations each of K = 1 to K = 14. To estimate the number of genetic clusters (K), we used *StructureSelector*^43^, and considered DeltaK^44^ and MedMeaK, MaxMeaK, MedMedK and MaxMedK^45^. A subpopulation is considered to belong to a cluster if its arithmetic mean (for MedMeaK and MaxMeaK) or its median (for MedMedK and MaxMedK) membership coefficient to that cluster is greater than a threshold value (set to 0.5^45^).

To estimate genetic diversity and mating system, we calculated observed heterozygosity and expected heterozygosity under Hardy–Weinberg equilibria, and F (inbreeding coefficient) in GenAlEx 6.51b^46^. To detect hybrids, we compared the genotypes of a subset of *E. melliodora* trees with other trees in the study site. Included in this analysis were 61 samples of *E. melliodora* selected randomly from all populations in the main dataset (except recruits) including natural stands, paddock trees, and planted trees, together with 42 samples of *E. albens*, and 11 samples from other species growing in close proximity including *E. crebra* and *E. blakelyi* (labelled “other spp.”). *Eucalyptus melliodora*, *E. albens* and *E. crebra* all belong in *Eucalyptus* Section *Adnataria* and are relatively likely to hybridise with each other^31,47^. *Eucalyptus blakelyi* is from Section *Exsertaria* and is less likely to hybridise with these species^31^. Loci were filtered as above (read depth of ≥ 10, a call rate of ≥ 0.9, a minor allele frequency of ≥ 0.01 and a reproducibility rate of ≥ 0.99), and a genetic cluster analysis was conducted in *ParallelStructure*. We used the admixture model of ancestry with correlated allele frequencies, a burn-in length of 80,000 followed by 120,000 MCMC replicates after burn-in, and we conducted 5 iterations each of K = 1 to K = 6. To estimate the number of genetic clusters (K), we used the Puechmaille method^45^ on *StructureSelector*^43^, which calculates MedMeaK, MaxMeaK, MedMedK and MaxMedK.

### Ethical approval

The experimental research and field studies on plants (either cultivated or wild), including the collection of plant material, complied with relevant institutional, national, and international guidelines and legislation. Samples were collected under NSW Scientific Licence SL102262.

## Results

### Population structure of *E. melliodora* trees

The DArTseq high-throughput microarray yielded 64,416 polymorphic SNP loci across the 221 samples. This data was of a high quality with 35.2% of loci at a call rate ≥ 85%, and an average call rate of 63.5%. A total of 219 individuals and 1406 loci were retained under our high stringency filters. Out of the 10 duplicate samples, the average similarity was 99.4% (9 duplicates had an average of 99.97% and one had an average of 94.3%). In all cases, the difference between the duplicates was due to a difference in the level of missing data (i.e. the number of loci that were scored), with no instances of loci being genotyped differently between the duplicates.

**Figure 2:**
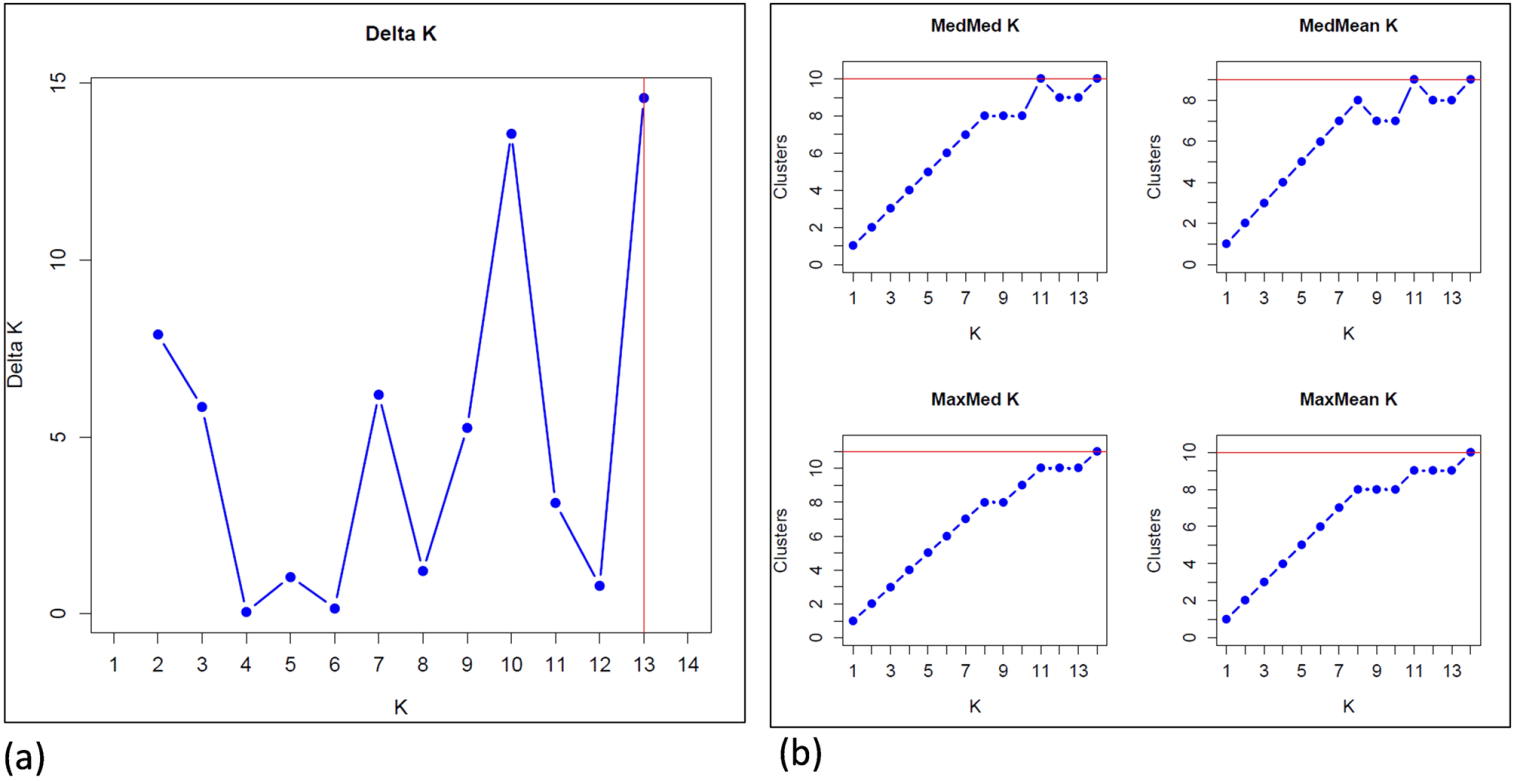
Number of genetic clusters (K) among the *E. melliodora* dataset based on 1406 loci estimated with (**a**) the Evanno method (DeltaK)^44^ and (**b**) the Puechmaille method^45^.

**Figure 3:**
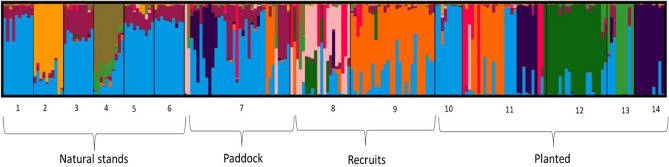
*Structure* plot from all populations of *E. melliodora* trees, including natural stands, paddock trees, recruits and planted trees, showing genetic clustering when K = 10.

In combination, the results of our pairwise *F*_*ST*_ analysis, PCA and Bayesian clustering analysis in *Structure* indicated that there was moderate genetic subdivision across the entire set of sampled *E. melliodora* trees. Global *F*_*ST*_ across all sites was 0.11, while pairwise *F*_*ST*_ values ranged from 0.01 to 0.32 and all were significantly different from zero (*p* < 0.05; Table 1). The number of genetic clusters (K) estimated from DeltaK, LnPK and MedMeaK, MaxMeaK, MedMedK and MaxMedK values was approximately 10 (Fig. 2). At the broadest level, the greatest amount of genetic differentiation was evident in the planted populations 12 and 14, some of the paddock trees and their respective recruits (Supplementary Figs. S2 and S3). Fine-scale population structure illustrated by the 10 genetic clusters in the *Structure* plot (Fig. 3) is described below.

**Table 1:** Pairwise *F*_*ST*_ comparisons between different populations of *E. melliodora* across the four groups of samples.

Population	Natural stands						Paddock trees	Recruits		Planted trees				
	1	2	3	4	5	6	7	8	9	10	11	12	13	14
1	0.000													
2	0.108	0.000												
3	0.051	0.136	0.000											
4	0.083	0.175	0.102	0.000										
5	0.048	0.126	0.078	0.098	0.000									
6	0.046	0.121	0.075	0.107	0.067	0.000								
7	0.020	0.099	0.040	0.067	0.041	0.036	0.000							
8	0.065	0.143	0.080	0.112	0.083	0.089	0.038	0.000						
9	0.078	0.149	0.094	0.126	0.104	0.100	0.059	0.095	0.000					
10	0.052	0.140	0.088	0.113	0.074	0.078	0.041	0.091	0.104	0.000				
11	0.029	0.114	0.047	0.081	0.055	0.052	0.011	0.047	0.031	0.056	0.000			
12	0.179	0.241	0.200	0.222	0.181	0.185	0.150	0.156	0.204	0.199	0.153	0.000		
13	0.071	0.170	0.107	0.133	0.101	0.096	0.061	0.104	0.124	0.099	0.076	0.217	0.000	
14	0.203	0.289	0.211	0.251	0.227	0.221	0.134	0.214	0.231	0.231	0.128	0.328	0.245	0.000

Fine-scale population differentiation illustrated by the 10 genetic clusters in the *Structure* plot showed that the six natural stands sampled from uncleared vegetation around the valley were moderately differentiated from one another (*F*_*ST*_ values in this group ranged from 0.02 to 0.17; Table 1). Four of these widely scattered stands formed a single undifferentiated cluster (dominated by light blue in Fig. 3; mean *F*_*ST*_ < 0.060) that was well represented within the paddock trees, while the remaining two natural stands (locations 2 and 4), were distinct and not represented among paddock trees, planted trees or recruits. The widely scattered paddock trees showed some differentiation from the natural stands (*F*_*ST*_ 0.029–0.097; Table 1) and also displayed remnant genotypes that were not present in the natural stands of the surrounding valley sides (Fig. 3).

The planted populations showed the highest level of differentiation from other groups (*F*_*ST*_ values ranged from 0.03 to 0.33; Table 1), and the fine-scale *Structure* plot identified six genetic clusters within this group, two of which were not represented elsewhere among the natural stands or paddock trees (populations 12 and 13, Fig. 3). Planted population 10 was genetically similar to the natural stands, while trees in population 11 appear to constitute a seed mix from the native stands and paddock trees (supported by low *F*_*ST*_ values between population 7 and 11; *F*_*ST*_ = 0.01, Table 1). Population 14 was the most differentiated population from all other populations (*F*_*ST*_ = 0.13–0.33; Table 1). However, the *Structure* plot suggests it is most closely related to some of the paddock trees (Fig. 3) which is supported by pairwise *F*_*ST*_ values, as populations 14 and 7 (paddock trees) have the lowest pairwise *F*_*ST*_ of all other comparisons with population 14 (*F*_*ST*_ = 0.13; Table 1).

All recruits were sampled near a pair of adult paddock trees, and they showed genotypic similarity to each of these pairs of paddock trees. Paddock trees R001 and R002 and all nearby recruits show some level of association with the maroon cluster, while paddock trees R077 and R078 and all nearby recruits showing some level of association with the dark green cluster (Fig. 3). In each case, only one other paddock tree showed a partial association with these clusters and there was no association with any of the sampled valley-side stands implying that recruits were the offspring of one or both of the neighbouring paddock trees (Fig. 3). Similarly, almost half of the recruits surrounding paddock trees R001 and R002 also showed a partial association with the navy-blue cluster which was almost unique to planted stand 12 located only 30 to 120 m from these recruits. Taken together, these data imply that planted individuals had successfully cross-pollinated with paddock trees and that most matings involved adult trees separated by only tens of metres.

### Genetic diversity and mating system among *E. melliodora* trees

The relictual paddock trees had the highest genetic diversity of any of the groups (mean *H*_*e*_ = 0.170; Table 2). The six natural stands were all similarly genetically diverse (mean *H*_*e*_ = 0.141; Table 2), but were less diverse than the paddock trees (Table 2). The planted trees had a similar level of genetic diversity to the natural stands (mean *H*_*e*_ = 0.141; Table 2; no significant difference p > 0.05, Students t-test), while the two populations of recruits had higher diversity than the planted and natural stands (mean *H*_*e*_ = 0.159).

**Table 2:** Genetic diversity measures of *E. melliodora* populations with standard errors.

Type	Population	N individuals	N loci	Ho ± SE	He ± SE	F_IS_
Natural	1	10	1406	0.113 ± 0.004	0.144 ± 0.004	0.258
	2	10	1406	0.127 ± 0.004	0.131 ± 0.005	0.079
	3	10	1406	0.122 ± 0.004	0.147 ± 0.004	0.211
	4	10	1406	0.112 ± 0.004	0.134 ± 0.004	0.208
	5	10	1406	0.118 ± 0.004	0.146 ± 0.004	0.234
	6	10	1406	0.116 ± 0.006	0.143 ± 0.005	0.231
	Mean			0.118 ± 0.004	0.141 ± 0.004	0.203
Paddock	7	34	1406	0.121 ± 0.003	0.170 ± 0.004	0.194
Recruits	8	30	1406	0.136 ± 0.004	0.161 ± 0.004	0.179
	9	28	1406	0.134 ± 0.004	0.158 ± 0.004	0.165
	Mean			0.135 ± 0.004	0.159 ± 0.004	0.172
Planted	10	9	1406	0.112 ± 0.004	0.136 ± 0.005	0.229
	11	28	1406	0.132 ± 0.004	0.173 ± 0.004	0.255
	12	19	1406	0.124 ± 0.003	0.129 ± 0.004	0.063
	13	10	1406	0.115 ± 0.004	0.134 ± 0.004	0.185
	14	10	1406	0.129 ± 0.004	0.132 ± 0.004	0.069
	Mean			0.122 ± 0.004	0.141 ± 0.004	0.160

*Ho* observed heterozygosity, *He* expected heterozygosity, *F*_*IS*_ inbreeding coefficient.

Most natural stands displayed heterozygous deficiencies consistent with a mixed mating system and low to moderate levels of inbreeding. Inbreeding coefficients (*F*_IS_*)* for the valley side stands averaged 0.203, while the relatively isolated paddock trees and surrounding recruits had mean inbreeding coefficients of 0.194 ± 0.004 and 0.172 ± 0.004 respectively (Table 2). The planted trees displayed inbreeding coefficients that ranged from 0.06 to 0.229 likely reflecting variable practices of seed collection but with an average inbreeding coefficient almost identical to the mean for the natural stands (Table 2).

### Detection of putative hybrids

**Figure 4:**
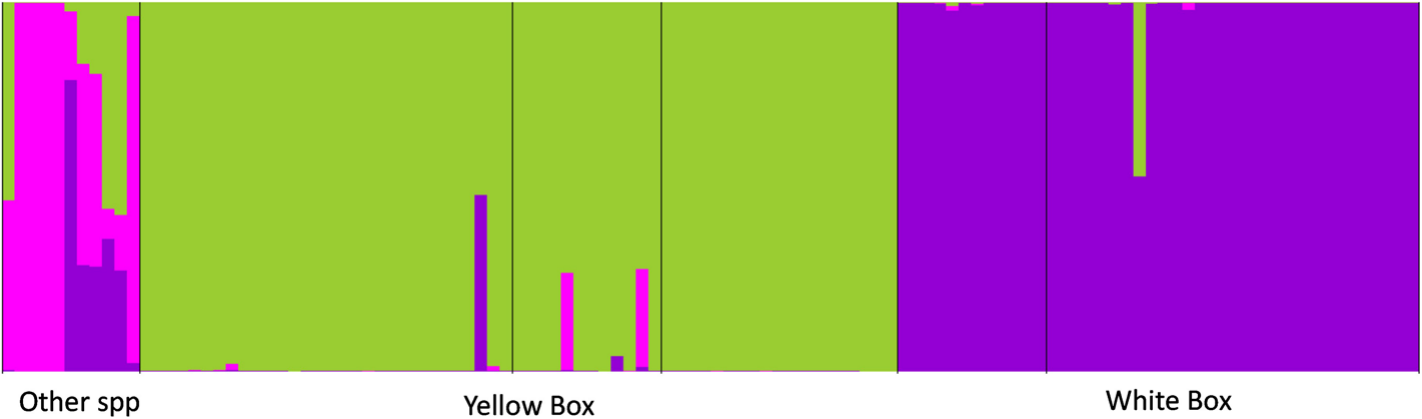
*Structure* plot showing K = 3 genetic clusters based on samples from 114 trees comprised of Yellow Box (*E. melliodora*), White Box (*E. albens*) and other eucalypt species.

After loci were filtered, genotypes were available for 1137 loci which comprised *E. melliodora* (n = 61), *E. albens* (n = 42) and individuals of other species (n = 11) representative of the seven common eucalypt species within the valley. The MedMeaK, MaxMeaK, MedMedK and MaxMedK values indicated that the most likely number of clusters was K = 3. Our analysis of these data using *ParallelStructure 2.3.4* showed that both the *E. melliodora* and *E. albens* trees formed clear clusters differentiated from the other species (Fig. 4). Hybridization was rare with four apparent hybrid individuals detected in the sample of *E. melliodora* and *E. albens* trees that we analysed. These comprised two *E. albens* X *melliodora* hybrids (one paddock tree and one from within a natural stand), one *E. melliodora* X other species hybrid and one *E. albens* X other species hybrid. Both of these were sampled from natural stands.

## Discussion

Our study of the population genetics of natural and revegetated *Eucalyptus melliodora* populations in WNP is one of the few studies that assess the genetics of woodland tree restoration by comparing the genetic structure of revegetated stands to both paddock trees and intact natural stands (e.g.^7^). We found that at a broad level, paddock trees and surrounding natural stands form comparably diverse and well-connected populations; moreover, the 30-year old Warrumbungle NP restoration program has been largely successful in adding genetically diverse stands derived from locally sourced seeds. In addition, our study further supports earlier work with both *E. melliodora* and other eucalypts in highlighting the value of paddock trees as both sources of existing genetic diversity and foci for recruitment^13,25,29^. This study builds on the work of Broadhurst^13^ who emphasised both the long and short-term value of paddock trees as reservoirs of genetic diversity and as potentially important pollen sources that minimise the effects of inbreeding within seed crops on planted trees. Our data show that paddock trees include genetic diversity not conserved elsewhere in the nearby stands, and that they were clearly parents of the majority of our sampled recruits. However, our data also show that similar to Broadhurst’s study^13^, a proportion of planted trees were not locally sourced and at least one paddock tree was a recent generation hybrid. Overall, our data suggest that the WNP Box-Gum Grassy Woodland restoration program has been particularly successful in capturing the genetic diversity of the broader population of *E. melliodora* within WNP, and the combination of this restoration and recruitment centred on paddock trees should ensure that the population retains diversity for future generations.

Contrary to our expectation based on Broadhurst^13^ that the revegetated (i.e., planted) individuals of *E. melliodora* would have lower genetic diversity than natural populations due to a low number of source individuals, we found that genetic diversity in the planted population was similar to the surrounding natural populations and the paddock trees, with the planted trees showing no reduction in genetic diversity. Other studies have shown varying results in restoration programs with some restoration populations having higher genetic diversity than reference sites, while others have lower diversity than reference sites. For example, a recent meta-analysis of 26 studies showed that levels of genetic diversity were strongly associated with the number of source sites used, and most revegetation projects that used multiple source sites showed higher genetic diversity than those that used only one site^48^. The comparable level of genetic diversity between the planted trees and the natural reference population in the surrounding bushland suggests that sourcing seed from six populations for a replanting program of this size would be sufficient to maintain genetic diversity in the revegetated population. From a conservation perspective, the genetic diversity of naturally occurring seedlings and the contribution of genetic material from remnant paddock trees provides stronger evidence of successful restoration. By extending the work of Broadhurst^13^ from looking at potential recruits (seeds), to established naturally occurring seedlings, we can have more confidence in our findings, since these plants have already survived local selective pressures and any genetic load inherited from interactions within and among planted and remnant trees.

As a group, the paddock trees were the most genetically complex and diverse trees of those sampled. The importance of paddock trees in maintaining ecosystem health and biodiversity within agricultural landscapes has been widely reported (reviewed in^25^ and^13^). Here, we demonstrate that they are a significant source of genetic variation which is important to capture in revegetation programs to increase adaptive potential, retain locally adapted genotypes, enable efficient selection, mitigate inbreeding, and minimise genetic drift^15,48–50^. The planted trees that were likely sourced from the paddock trees showed similar levels of genetic diversity to natural stands, suggesting that seed collected from paddock trees is of sufficient quality to contribute to population regeneration, as also identified in other studies^25,30^.

Within WNP, we found that the natural populations of *E. melliodora*, including the paddock trees, showed some genetic differentiation suggesting that while historic gene flow has been sufficient to maintain strong connections among some stands, it has not prevented the development of some local population differentiation. The greatest level of population subdivision across the study site was among the planted trees; however, this reflects patterns of seed collection for the plantings rather than natural levels of connectivity. Our results showed quite high *F*_*ST*_ values in comparison to earlier studies^51,52^ that found little differentiation of *E. melliodora* populations spread across much of its geographic range (*F*_*ST*_ = 0.029, 0.04 resp. cf. 0.11 in this study). Our study is one of the few studies to use DArT-seq markers in a population genetic study of eucalypts (but see^53,54^). These studies also found relatively high levels of structure within species (maxima ~ 0.41 for *E. microcarpa* and 0.11 for *E. cunninghamii*, [^53,54^ resp.]). The use of DArT-seq markers, primarily due to the generation of large numbers of SNPs across the genome, appears to improve population differentiation^55^, but caution is advised in comparisons with *F* statistics derived from other markers^55^.

Bayesian clustering analysis detected a number of unique genetic clusters within the paddock trees which suggests that some genotypes may be favoured within the valley floor rather than valley side populations. Planted population 14 and the relictual paddock tree from which it appears to have been seeded, showed the greatest level of genetic differentiation from other populations, which could indicate that selection is driving micro-site adaptation on the valley floor. A previous study of population genetic differentiation in *E. obliqua* found that local adaptation in response to different elevations can occur over as little as a few hundred meters^56^. This in turn might imply that it is important to maintain strict local provenance in attempting restoration, to maintain site-specific adaptations. However, given that at least some trees have recruited since the clearing associated with pastoral activity, it is also possible that recruits derived from the isolated relictual paddock trees are simply the result of more erratic patterns of long-distance pollination than would occur in valley side stands. Most pollen dispersal in *E. melliodora* and other eucalypts is thought to occur over tens of metres, although intraspecific and interspecific pollination over several kms has been inferred for a range of eucalypt species^17,28,32,57^ including *E. melliodora*^13^*.*

Our genetic cluster analyses indicated that the planted trees were sourced from at least six different populations, and confirmed that a mixed provenancing strategy was used in this revegetation program. Planted stands 10, 11 and 14 were sourced from local seed from paddock trees and possibly surrounding natural stands, while populations 12 and 13 were probably not sourced locally. There is strong evidence that some of the revegetated trees planted in close proximity to paddock trees have interbred with these paddock trees to contribute to natural recruitment. There is increasing support for adopting strategies of mixed provenancing (i.e., using seed from multiple source sites in revegetation programs) to broaden the genetic base of revegetated areas^48,53^ in order to improve ecological outcomes and increase adaptive potential to environmental change^49,58^. However, the issue of micro-site genetic variation and adaptation (identified above), also presents a challenge for mixed provenance strategies.

Elevated inbreeding with the consequent risk of inbreeding depression are threats to the long-term survival of both isolated plants and stands restored from small numbers of individuals^59^. However, in this study neither the (often isolated) paddock trees, planted trees or the two sampled stands of recruits showed evidence that inbreeding levels were higher than those seen in the six natural stands. It is noteworthy, however, that Broadhurst^13^, while acknowledging that *E. melliodora* has a mixed mating system, reported an outcrossing rate (*t*) estimated through maternal adult/seed comparisons that was consistently close to 1.0. In contrast, the genotypic make-up of both adult and recruit stands in this study suggests that all sampled WNP populations were more inbred. Estimation of *t* assuming a stable equilibrium within our sampled populations ranged from ~ 0.7 to 0.8 (where *t* at equilibrium = (1 − F)/(I + F)^60^).

Perhaps surprisingly, given the relatively low outcrossing rates of the WNP stands and the apparent isolation of the paddock trees, we detected little evidence of hybridisation across the trees sampled in our study. The four recent inter-specific hybrids that we detected were within the scattered paddock trees or natural stands and likely represent a cohort of trees that pre-date agricultural clearing, suggesting historic or even pre-colonial hybridisation. Hybrids among *E. melliodora* and other members of *Eucalyptus* section *Adnataria* are common [47 and references therein]. Section *Adnataria* also includes *E. albens*, *E. crebra* and *E. sideroxylon*, all of which are common within study area and have long and overlapping flowering seasons. The restoration plantings have increased connectivity and relative abundance of multi-species pollen in the restoration area and may increase the likelihood of hybridisation^16,17,32^. An assessment of naturally occurring offspring would provide insight into the significance of this phenomenon for restoration practice.

In conclusion, the results of this study indicate that revegetated populations of *E. melliodora* in WNP are largely comprised of genetically diverse, mostly local provenance trees. The high genetic diversity and multiple seed-sources evident in the planted populations are testament to the success of this component of the ecological restoration project. Relictual paddock trees have been an important source of historic genetic diversity for this restoration program, and these trees can continue to be an important source of new recruits in the revegetated area and a valuable source of genetic diversity for restoration programs.

## Supplementary Information

Supplementary Information.

## Data Availability

The dataset generated during and/or analysed during the current study has been placed in the European Variation Archive (EVA) as project PRJEB59174. It is also in the Dryad repository https://datadryad.org with https://doi.org/10.5061/dryad.hx3ffbgh1.
