# Supplementary material for: Genetic assessment of the value of restoration planting within an endangered eucalypt woodland
Source: Sci Rep. 2023 Apr 21;13:6583. doi: 10.1038/s41598-023-33720-z (PMC10121665; doi:10.1038/s41598-023-33720-z)
Supplement: Supplementary file 1 — Supplementary Information. [file 41598_2023_33720_MOESM1_ESM.pdf]

Supplementary material for:

Genetic assessment of the value of restoration planting within an endangered eucalypt woodland

Natalie L. Rosser, Anthony Quinton, Huw Davey, David J. Ayre and Andrew J. Denham

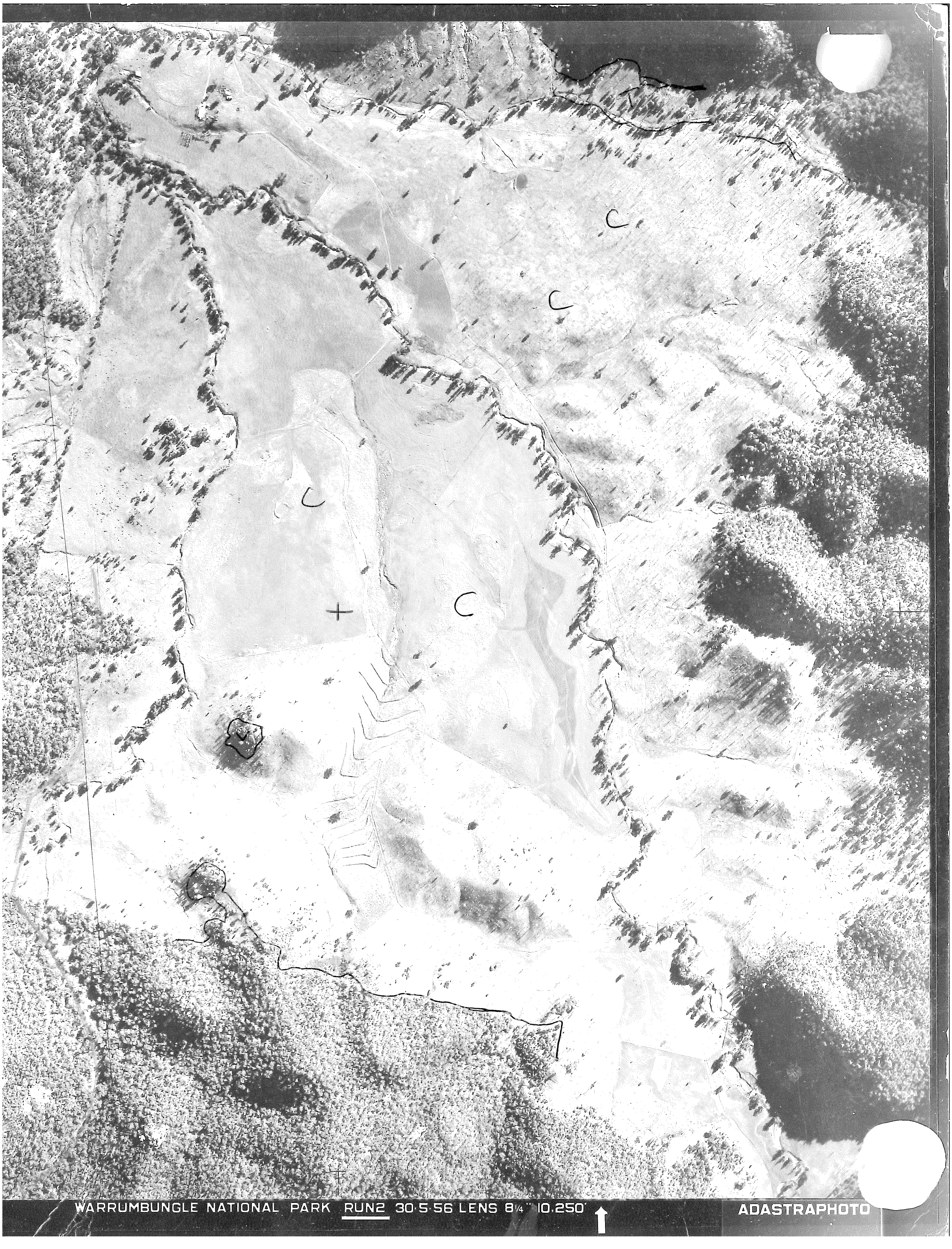

Supplementary Fig. S1 Aerial photograph from 1956 used to locate surviving paddock trees in the central valley of Warrumbungle National Park. The annotations present are also historical. Photograph reproduced with permission of NSW National Parks and Wildlife Service

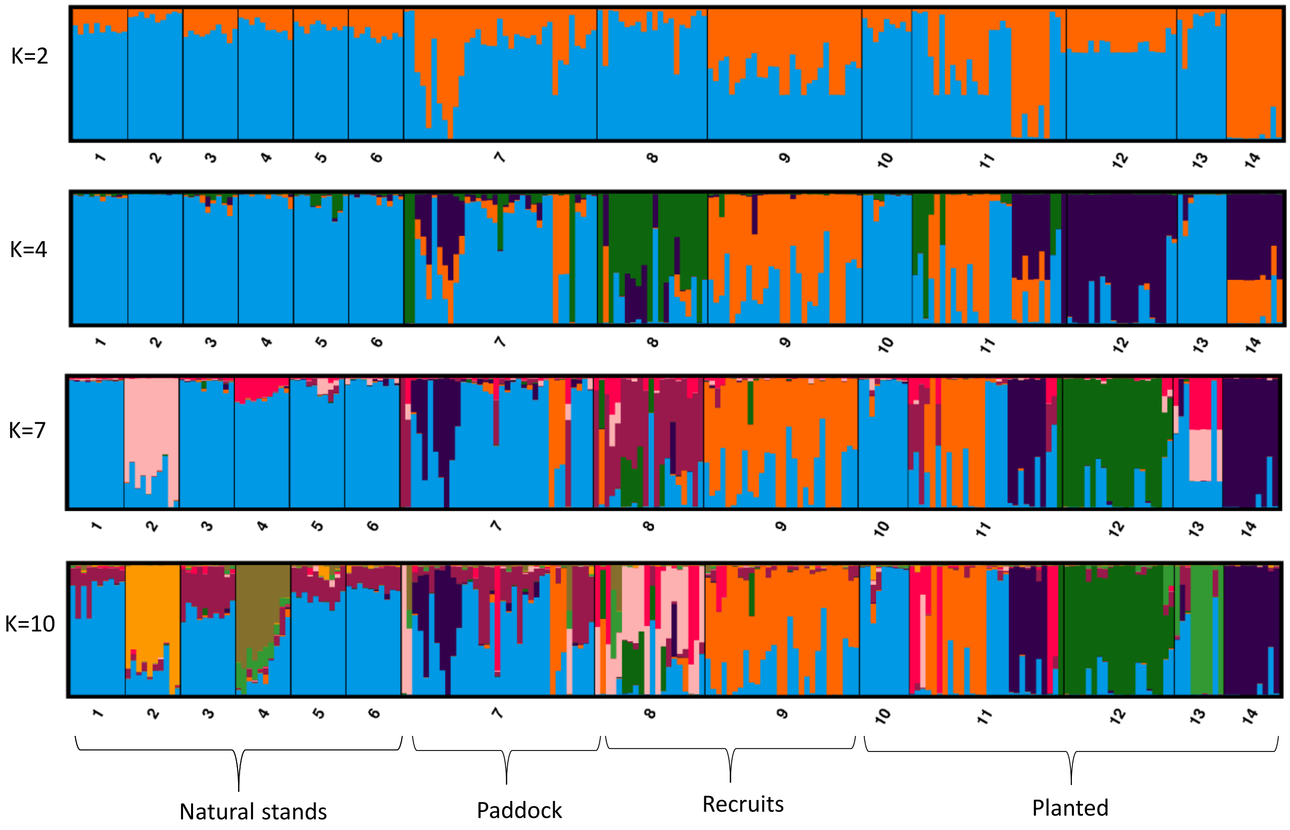

Supplementary Fig. S2 Structure plots showing genetic clusters (K) with values of K=2, 4,7 and 10

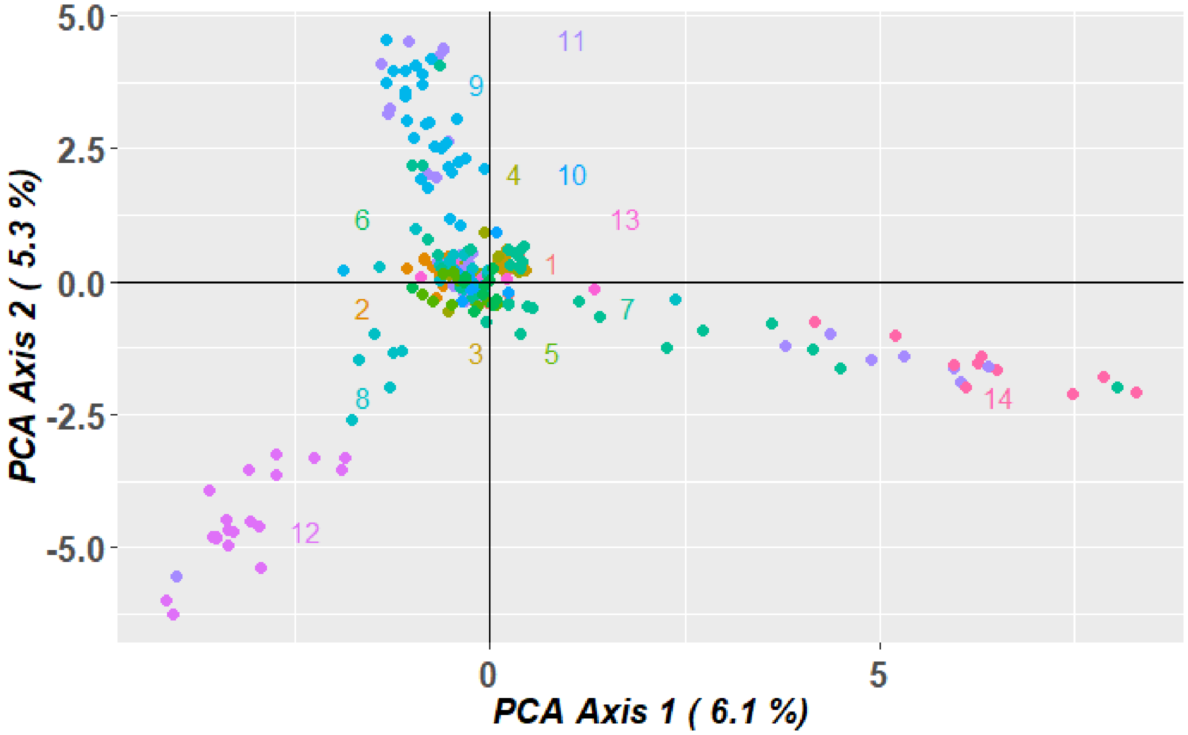

Supplementary Fig. S3 PCA ordination showing the contribution of two principal component axes to the genetic separation of *E. melliodora* samples. Colours and population numbers as per Fig. S2. Populations 1-6, Natural stands; Population 7, Paddock trees; Populations 8 and 9, Recruits; Populations 10-14, Planted trees
